# Aboriginal status is a prognostic factor for mortality among antiretroviral naïve HIV-positive individuals first initiating HAART

**DOI:** 10.1186/1742-6405-3-14

**Published:** 2006-05-24

**Authors:** Viviane D Lima, Patricia Kretz, Anita Palepu, Simon Bonner, Thomas Kerr, David Moore, Mark Daniel, Julio SG Montaner, Robert S Hogg

**Affiliations:** 1 British Columbia Centre for Excellence in HIV/AIDS, St. Paul's Hospital; Vancouver, Canada; 2 Departments of Medicine and/or Healthcare and Epidemiology, Faculty of Medicine, University of British Columbia, Vancouver, Canada; 3 Département de médecine sociale et préventive, Université de Montréal et Centre de recherche du Centre hospitalier de l'Université de Montréal, Québec, Canada

## Abstract

**Background:**

Although the impact of Aboriginal status on HIV incidence, HIV disease progression, and access to treatment has been investigated previously, little is known about the relationship between Aboriginal ethnicity and outcomes associated with highly active antiretroviral therapy (HAART). We undertook the present analysis to determine if Aboriginal and non-Aboriginal persons respond differently to HAART by measuring HIV plasma viral load response, CD4 cell response and time to all-cause mortality.

**Methods:**

A population-based analysis of a cohort of antiretroviral therapy naïve HIV-positive Aboriginal men and women 18 years or older in British Columbia, Canada. Participants were antiretroviral therapy naïve, initiated triple combination therapy between August 1, 1996 and September 30, 1999. Participants had to complete a baseline questionnaire as well as have at least two follow-up CD4 and HIV plasma viral load measures. The primary endpoints were CD4 and HIV plasma viral load response and all cause mortality. Cox proportional hazards models were used to determine the association between Aboriginal status and CD4 cell response, HIV plasma viral load response and all-cause mortality while controlling for several confounder variables.

**Results:**

A total of 622 participants met the study criteria. Aboriginal status was significantly associated with no AIDS diagnosis at baseline (p = 0.0296), having protease inhibitor in the first therapy (p = 0.0209), lower baseline HIV plasma viral load (p < 0.001), less experienced HIV physicians (P = 0.0133), history of IDU (p < 0.001), not completing high school (p = 0.0046), and an income of less than $10,000 per year (p = 0.0115). Cox proportional hazards models controlling for clinical characteristics found that Aboriginal status had an increased hazard of mortality (HR = 3.12, 95% CI: 1.77–5.48) but did not with HIV plasma viral load response (HR = 1.15, 95% CI: 0.89–1.48) or CD4 cell response (HR = 0.95, 95% CI: 0.73–1.23).

**Conclusion:**

Our study demonstrates that HIV-infected Aboriginal persons accessing HAART had similar HIV treatment response as non-Aboriginal persons but have a shorter survival. This study highlights the need for continued research on medical interventions and behavioural changes among HIV-infected Aboriginal and other marginalized populations.

## Introduction

There are trends for global health concerns to have a greater impact on ethnic minorities [1-5]. For example, the human immunodefeciency virus (HIV) epidemic has disproportionately affected Aboriginal persons throughout North America. American Indians and Alaska Natives make up 6% of all new HIV infections in the USA, yet they represent less than 1 percent of the population [6]. Studies attempting to explain this disparity have shown that both Aboriginal men and women are at an increased risk of antecedent risk factors for HIV infection including sexual abuse, a history of IDU, poverty, poor mental health and involvement in the sex trade [7-9] Furthermore, within risk groups, for example amongst individuals with a history of injection drug use (IDU), Aboriginal men [10,11] and women [11,12] are at a higher risk of being infected with HIV.

In addition to a higher risk of HIV infection, it has been shown that Aboriginal persons were more likely to suffer from other morbidities that can complicate HIV disease progression. For example, HIV-infected Aboriginal youths, were more likely to be co-infected with hepatitis C virus [13-15], being depressed [16,17], and having anemia [18], all of which have been independently associated with morbidity or mortality in HIV-infected individuals [19-21].

Further disparity for Aboriginal persons and other marginalized groups is found when considering access to treatment for HIV. In the USA, assessment of state surveillance and claims data revealed that access to HIV therapy was influenced by state policies which showed racial inequality in pharmaceutical access [22]. In British Columbia, Canada, where antiretrovirals are distributed at no cost, Aboriginal ethnicity was negatively associated with receiving HIV treatment before death [23] and positively associated with leaving the hospital against medical advice [24].

Although the impact of Aboriginal status on HIV incidence, HIV disease progression, and access to treatment has been investigated previously, little is known about the relationship between Aboriginal ethnicity and outcomes associated with highly active antiretroviral therapy (HAART). There have been studies on racial/ethnic (black, white and Hispanic) differences in response to HAART [25-28], yet, to our knowledge, there has been only one small study examining this issue with Aboriginal persons in Greenland [29], but none including Aboriginal persons in urban areas. Therefore, the present study examined whether Aboriginal and non-Aboriginal persons respond differently to HAART by measuring HIV plasma viral load response, CD4 cell response and time to all-cause mortality. We also examined potential confounder effects of various demographic and clinical characteristics on the association between Aboriginal ethnicity and HIV disease progression.

## Methods

### HIV/AIDS drug treatment program

The distribution and the population-based monitoring of antiretroviral therapy in British Columbia have been extensively described in the literature [30-32]. The Centre distributes antiretroviral drugs based on guidelines generated by the Therapeutic Guidelines Committee, which is made-up of physicians, pharmacists, virologists, health service researchers and economists. The Providence Health Care Ethics Committee for Human Experimentation has approved use of the data generated from the program for research purposes.

### Data collection

All antiretroviral treatment recipients in the province are entered into an Oracle-based monitoring and evaluation reporting system that uses standardized indicators to prospectively tract the antiretroviral use and clinical and health status of HIV-1 positive individuals. Physicians enrolling an HIV-1 infected individual into the system must complete a drug request enrolment prescription form, which compiles information on the applicant's address, past HIV-specific drug history, CD4 cell counts, plasma HIV-1 RNA, current drug requests, and enrolling physician data. Typically, persons receiving antiretroviral therapy are monitored by physicians at intervals no longer than three months at which time prescriptions are renewed or modified. At the time of the initial dispensation, participants are asked to provide informed consent for accessing medical electronic records (which may be used for health utilization studies, but is not relevant to the analyses in this study), and complete a participant survey, which elicits information on socio-demographic characteristics, clinical and health status, and alternative therapy use. Both the consent form and the participant survey are optional and participant's refusal to do either will not limit his or her access to free antiretroviral therapy. At the same time, the treating physicians are asked to complete a clinical staging form using the World Health Organization (WHO) clinical staging system.

The Centre recommends that plasma HIV plasma viral load levels and CD4 cell counts be monitored at baseline (time of enrolment), at four weeks after starting antiretroviral therapy and every three months thereafter. HIV plasma viral load were determined using the Roche Amplicor Monitor assay (Roche Diagnostics, Laval, Quebec, Canada) using either the standard method or the ultrasensitive adaptation. CD4 cell counts were measured by flow cytometry, followed by fluorescent monoclonal antibody analysis (Beckman Coulter, Inc., Mississauga, Ontario, Canada).

### Study participants

All HIV-infected men and women in the current study were entered into the Centre's monitoring and evaluation system when they were first prescribed antiretroviral agents. Eligible study participants are persons who were ≥18 years old, naïve to antiretroviral therapy when they started HAART, and first dispensed triple combination therapy consisting of two nucleoside reverse transcriptase inhibitors and either a protease inhibitor (unboosted) or a non-nucleoside reverse transcriptase inhibitor, between August 1, 1996 and September 30, 1999. Eligible participants must have completed at least one questionnaire and undertaken at least two follow-up CD4 cell count and HIV plasma viral load tests.

### Outcome measures and explanatory variables

There were three primary endpoints of interest in this study: (1) time to HIV plasma viral load suppression as measured from the time of starting HAART to the first of two consecutive plasma HIV viral loads of < 500 copies/ml; (2) CD4 cell response as measured by the time of starting HAART to the first CD4 cell test with an increase of 100 cells over baseline; and (3) all cause mortality.

Event-free subjects were right censored as of June 30, 2003. Participants included in this analysis were not followed after this date and those lost to follow-up were censored at the date of last known contact with the HIV/AIDS Drug Treatment Program. For the first outcome, individuals we also censored if the last available HIV plasma viral load test result was the first to be < 500 copies/ml, and the censoring was applied at the time of the prior test. Deaths occurring during the follow-up period were identified on a continuous basis from physician reports and through annual record linkages carried out with the British Columbia Division of Vital Statistics.

The predictor variable of interest was Aboriginal status. A number of potential confounders have been considered: baseline CD4 cell count, baseline plasma HIV viral load, physician's experience with HIV-infected patients, gender, age, income, education, AIDS diagnosis at baseline, protease inhibitor use, year of initiation of therapy, history of IDU and adherence. Physician experience was estimated for the first follow-up physician of each patient. It was defined as the cumulative number of HIV-infected patients receiving antiretroviral therapy within their practice, by the date of subject's first known eligibility [33]. In all analysis, in order to get a more parsimonious fit we transformed this variable by dividing the original value by 10. Income was defined as an average yearly income of ≥$10,000 or <$10,000 (in Canadian dollars). Education was defined as having completed high school. History of IDU was defined as "ever-injected drugs" (yes or no), which was physician or self-reported. Adherence was estimated using pharmacy refill compliance. In brief, we divided the number of months of HAART dispensed by the number of months of follow-up in the first year of therapy. This measure of adherence has been found to be independently associated with HIV viral suppression and survival among HIV-infected persons enrolled in the HIV/AIDS Drug Treatment Program [34,35]. Patients were defined a priori as non-adherent if they received antiretroviral medications for less than 95% of the follow-up period during the first year of therapy, as in previously published work [36,37].

### Statistical analyses

Cox-proportional hazard regression was used to model the effect of Aboriginal status and other potential confounders on the time to virologic suppression, time to CD4 response and survival time [38]. Categorical variables were analyzed using Pearson chi squared statistics, normally distributed continuous variables were analyzed using t-tests for independent samples, and skewed continuous variables were analyzed using Wilcoxon rank sum tests. The assumptions of proportional hazards were examined graphically.

A number of potential confounders have been included in our analyses. In the selection of important confounders, we applied a method based on the magnitude of change in the coefficient of Aboriginal status [39,40]. Age, sex and adherence were forced into all models. A stepwise approach was employed to select additional confounders [41]. Starting with the full model, variables were dropped one at a time, using the relative change in the coefficient for Aboriginal status as a criterion, until the maximum change from the full model exceeded 5%, which is a more conservative approach than the one suggested by Maldonado and Greenland [39].

Analyses were performed using SAS software version 8.02 (SAS, Cary, NC). All tests of significance were 2-sided, with a *P *value of less than 0.05 indicating that an association was statistically significant.

## Results

Between August 1st, 1996 and September 30, 1999, a total of 1,312 antiretroviral naïve participants aged 18 years and over initiated triple combination therapy consisting of two nucleosides plus a protease-inhibitor or a non-nucleoside reverse transcriptase inhibitor. Of these, 121 (9.2%) were excluded in this analysis for not having both baseline CD4 and plasma HIV-1 RNA levels measures available within six months prior to the start of antiretroviral therapy or having initiated therapy as part of a clinical trial. Among the remaining 1,191 participants a total of 622 individuals (560 men and 62 women) had reported their aboriginal status.

Study participants were very heterogeneous when compared to those excluded from the analysis. Participants in the study: (1) enrolled mainly in 1996–1997 whilst non-participants enrolled mainly in 1998–1999 (57% versus 63%; p-value: < 0.001); (2) were older (median age 36.3 versus 37.9 years; p-value 0.0003); (3) had lower baseline CD4 cell count (median CD4 cell count 260 versus 290 cells/mm3; p-value: 0.0091); (4) had higher adherence than non-participants (71% versus 42%; p-value: < 0.0001); (5) were treated by more experienced physicians (median experience 55 versus 37 patients; p-value: 0.0003). No other differences were noted between the two groups.

At baseline, for all participants, the median age was 38 years (interquartile range (IQR): 33–44 years), CD4 cell count was 260 cells/mm3 (IQR: 110–410 cells/mm3), and HIV plasma viral load was 130,000 copies/ml (IQR: 42,000–320,000). A total of 50.0% of participants had an average annual income of less than $10,000 dollars, 38.9% did not complete high school, 34.2% had a history of IDU, 17.2% had an AIDS diagnosis, and 14.0%, 41.3%, 24.1% and 20.6%, respectively, started therapy in 1996, 1997, 1998 and 1999. At baseline, 77% initiated a protease inhibitor based regime as compared to a non-nucleoside reverse transcriptase regime. During the first year of follow-up 29.4% of participants had adherence level less than 95%.

Most (N = 568; 91.3%) participants achieved 2 consecutive plasma HIV viral loads of < 500 copies/ml before the end of study. Median time to suppression among the 568 participants was 3.4 months (IQR: 1.8–7.3 months), and 3.6 months (IQR: 1.8–9.0 months) among all patients. Similarly, 560 (90.0%) of participants achieved a CD4 cell recovery of 100 cells over baseline during follow-up period. Median time to recovery among the 560 participants was 5.0 months (IQR: 1.9–12.3 months), and was 6.0 months (IQR: 2.0–16.1 months) among all patients. As of June 30, 2003, a total of 67 deaths were identified in the study population over the follow-up period with an overall crude mortality rate of 10.8%. Among all causes of death, HIV disease resulting in infectious and parasitic diseases (excluding acute HIV infection syndrome) (N = 32, 47.8%) and injuries due to intentional self-harm (N = 13, 19.4%) were responsible for over 67% of all deaths. The remaining deaths (N = 22, 33%) were attributable to a total of twelve causes of deaths. The overall median time of follow-up was 62.5 months (inter quartile range: 50.3, 71.8 months).

Among the 622 individuals, 88 (14.1%) described themselves as being Aboriginal. As noted in Table 1, 8 (9.1%) commenced therapy in 1996, 32 (36.4%) in 1997, 21 (23.9%) in 1998, and 27 (30.7%) in 1999. Over half of these participants (59; 67.0%) initiated therapy with a protease-inhibitor, while the rest of the study participants (29; 33.0%) had a regimen that included a non-nucleoside reverse transcriptase inhibitor. Aboriginal status was associated with a history of injection drug use, not completing high school, an annual income < $10,000, not having an AIDS diagnosis at baseline, protease inhibitor use, having lower baseline plasma HIV viral load, and less experienced HIV physicians (p < 0.05). Age, gender, year of initiation of therapy, baseline CD4 cell count, and adherence were not significantly associated with being Aboriginal.

**Table 1:** A comparison of baseline socioeconomic and clinical characteristics of Aboriginal and non-Aboriginal participants

Variable	Aboriginal	Not Aboriginal	P-value
	(n = 88)	(n = 534)	
**Year of initiation of therapy, no. (%)**			
1996	8 (9)	79 (15)	0.0588
1997	32 (36)	225 (42)	
1998	21 (24)	129 (24)	
1999	27 (31)	101 (19)	
**Gender, no. (%)**			
Female	13 (15)	49 (9)	0.1044
Male	75 (85)	485 (91)	
**Age**			
Median	37	38	0.9669
Interquartile range	33 – 45	33 – 44	
**AIDS diagnosis, no. (%)**			
Yes	8 (9)	99 (19)	0.0296
No	80 (91)	435 (81)	
**Protease inhibitor use**			
Yes	59 (67)	418 (78)	0.0209
No	29 (33)	116 (22)	
**Baseline CD4 cell count (cells/mm3)**			
Median	275	260	0.6551
Interquartile range	150 – 425	110 – 410	
**Baseline plasma HIV viral load (copies/ml)**			
Median	89,350	140,000	< 0.001
Interquartile range	21, 750 – 185,000	45,000 – 340, 000	
**Adherence (<95%), no. (%)**			
Yes	58 (66)	381 (71)	0.2995
No	30 (34)	153 (29)	
**HIV physician experience**			
Median	29	60	0.0133
Interquartile range	3 – 116	6 – 166	
**History of IDU, no. (%)**			
Yes	52 (59)	161 (30)	< 0.001
No	36 (41)	373 (70)	
**Completed high-school, no. (%)**			
Yes	38 (43)	298 (56)	0.0046
No	43 (49)	171 (32)	
Missing	7 (8)	65 (12)	
**Income, no. (%)**			
< $10,000	49 (58)	211 (43)	0.0115
>= $10,000	36 (42)	281 (57)	
Missing	3 (3)	42 (8)	

Table 2 shows the univariate and multivariate associations of aboriginal status, clinical and socio-demographic confounders with time to the first CD4 cell test with an increase of 100 cells over baseline. Aboriginal status was not associated with CD4 cell count response, but only baseline HIV plasma viral load, age, physician experience and adherence.

**Table 2:** Univariate and Multivariate Cox proportional hazard models examining the association between being Aboriginal and time to a CD4 cell increase of 100 above baseline

Variable	Unadjusted HR	Adjusted HR
	(95% CI)	(95% CI)
Aboriginal (Yes versus No)	0.85 (0.65, 1.11)	0.95 (0.73, 1.23)
Plasma HIV viral load (per log 10 increase)	1.21 (1.06, 1.38)	1.27 (1.12, 1.44)
CD4 cell count (per 100 decrease)	0.96 (0.92, 1.00)	--
Gender (Female versus Male)	1.85 (1.30, 2.64)	1.28 (0.94, 1.74)
Age	1.00 (0.99, 1.01)	0.99 (0.98, 1.00)
Physician experience (per 10 patients)	1.03 (1.02, 1.04)	1.01 (1.01, 1.02)
Completed high-school (Yes versus No)	1.03 (0.86, 1.24)	--
Income (<$10,000 versus ≥ $10,000)	0.83 (0.68, 1.00)	0.97 (0.81, 1.17)
Baseline Combination (PI versus NNRTI)	0.81 (0.66, 1.01)	0.79 (0.62, 1.01)
Adherence (>= 95% versus <95%)	1.73 (1.39, 2.14)	1.57 (1.28, 1.94)
AIDS diagnosis (Yes versus No)	0.96 (0.75, 1.22)	--
History of IDU (Yes versus No)	0.79 (0.65, 0.96)	0.92 (0.75, 1.13)
Year of initiation of therapy		
1996	1.00 (--)	1.00 (--)
1997	0.95 (0.72, 1.26)	1.07 (0.81, 1.40)
1998	0.94 (0.69, 1.27)	1.14 (0.84, 1.55)
1999	1.03 (0.75, 1.40)	1.10 (0.78, 1.55)

**Notes: **The symbol – means that variable was not included in the analysis

Table 3 shows the results for the univariate and multivariate associations of Aboriginal status, clinical and socio-demographic confounders with time to the first of two consecutive plasma HIV viral loads of < 500 copies/ml. In these analyses Aboriginal status was not associated with HIV plasma viral load response. The multivariate analysis shows that only baseline HIV plasma viral load, age, education, adherence, and history of IDU were associated with HIV plasma viral load response.

**Table 3:** Univariate and Multivariate Cox proportional hazard models examining the association between being Aboriginal and time to the first of two consecutive plasma HIV viral loads of < 500 copies/ml

Variable	Unadjusted HR	Adjusted HR
	(95% CI)	(95% CI)
Aboriginal (Yes versus No)	0.90 (0.69, 1.17)	1.15 (0.89, 1.48)
Plasma HIV viral load (per log 10 increase)	0.73 (0.65, 0.81)	0.73 (0.65, 0.82)
CD4 cell count (per 100 decrease)	0.98 (0.93, 1.02)	--
Gender (Female versus Male)	1.25 (0.92, 1.70)	1.09 (0.81, 1.48)
Age	1.01 (1.00, 1.02)	1.01 (1.00, 1.02)
Physician experience (per 10 patients)	1.02 (1.01, 1.03)	--
Completed high-school (Yes versus No)	1.64 (1.37, 1.97)	1.37 (1.14, 1.65)
Income (<$10,000 versus ≥ $10,000)	0.66 (0.55, 0.79)	0.90 (0.75, 1.09)
Baseline Combination (PI versus NNRTI)	0.55 (0.45, 0.68)	--
Adherence (>= 95% versus <95%)	4.21 (3.32, 5.34)	3.46 (2.77, 4.31)
AIDS diagnosis (Yes versus No)	1.14 (0.91, 1.44)	--
History of IDU (Yes versus No)	0.56 (0.46, 0.68)	0.74 (0.60, 0.92)
Year of initiation of therapy		
1996	1.00 (--)	1.00 (--)
1997	0.95 (0.73, 1.23)	1.16 (0.89, 1.51)
1998	1.02 (0.77, 1.35)	1.49 (1.10, 2.00)
1999	1.06 (0.79, 1.41)	1.79 (1.31, 2.43)

**Notes: **The symbol – means that variable was not included in the analysis

The univariate and multivariate associations of aboriginal status, clinical and socio-demographic confounders with time to death are displayed in Table 4. Aboriginal status was strongly associated with mortality in both univariate (hazard ratio (HR) = 2.87, 95% confidence interval (CI): 1.70–4.84) and multivariate analyses (HR = 3.12, 95%CI: 1.77–5.48). In addition to Aboriginal status, the multivariate analysis shows that age, income, and adherence were also associated with mortality.

**Table 4:** Univariate and multivariate Cox proportional hazard models examining the association between Aboriginal status and mortality

Variable	Unadjusted HR	Adjusted HR
	(95% CI)	(95% CI)
Aboriginal (Yes versus No)	2.87 (1.70, 4.84)	3.12 (1.77, 5.48)
Plasma HIV viral load (per log 10 increase)	1.27 (0.86, 1.86)	--
CD4 cell count (per 100 decrease)	1.10 (0.97, 1.25)	--
Gender (Female versus Male)	0.89 (0.41, 1.95)	0.98 (0.41, 2.34)
Age	1.04 (1.01, 1.07)	1.06 (1.03, 1.09)
Physician experience (per 10 patients)	0.99 (0.96, 1.01)	--
Completed high-school (Yes versus No)	0.63 (0.39, 1.02)	--
Income (<$10,000 versus ≥ $10,000)	2.76 (1.59, 4.82)	1.86 (1.03, 3.34)
Baseline Combination (PI versus NNRTI)	1.22 (0.65, 2.29)	1.65 (0.82, 3.31)
Adherence (>= 95% versus <95%)	0.32 (0.20, 0.52)	0.41 (0.24, 0.71)
AIDS diagnosis (Yes versus No)	1.59 (0.91, 2.79)	--
History of IDU (Yes versus No)	2.18 (1.35, 3.51)	1.47 (0.82, 2.62)
Year of initiation of therapy		
1996	1.00 (--)	1.00 (--)
1997	0.49 (0.27, 0.92)	--
1998	0.67 (0.34, 1.32)	--
1999	0.34 (0.14, 0.82)	--

**Notes: **The symbol – means that variable was not included in the analysis

## Discussion

We found that there is no significant difference between HIV-infected Aboriginal persons and non-Aboriginal persons regarding the time to HIV plasma viral load suppression of < 500 copies/ml and time to CD4 cell response of 100 cells over baseline after initiation of HAART. There was however, a significant higher mortality risk for Aboriginal persons after the initiation of HAART. After adjustment for confounder variables, Aboriginal persons had mortality rates 3.12 times higher than non-Aboriginal persons. It is interesting to note that the only clinical characteristic associated with mortality risk in this population was adherence during first year of follow-up. The other variables associated with mortality were socio-demographic characteristics of the participants (age and income). We observed that clinical factors were only a significant predictor when we looked at virologic and immunologic responses. Note that among the clinical factors, poor adherence was the strongest predictor of adverse outcomes in all analyses. To explain this paradox, we considered potential confounding effects, given that race/ethnicity are closely intertwined with socioeconomic status and behavioural factors [1,4]. Our method of selecting confounding factors ensured that the final models were parsimonious while at the same time the estimates were not substantially affected by any potential confounders available in the data.

Other studies have examined the effect of ethnicity on response to HAART but to our knowledge, this was the first study to specifically examine this issue for Aboriginal persons living in large urban areas. Prior studies looked at potential differences among ethnic groups in response to HAART by measuring short-term virologic and immunologic response or differences in survival. In a Danish cohort, race (white versus not white) did not predict differences in virologic suppression and CD4 cell response one year after initiating HAART [27]. Race (white versus not white) also did not independently predict virologic response or CD4 response in a group of American men who have sex with men after 33 months of initiating HAART [28]. Another study also found that race (Hispanic, Black or White) was not a factor in CD4 cell count response among American patients who experienced plasma HIV viral suppression within 6 months of initiation of HAART [26]. In a comparison between an African and a European cohort both on HAART there were also no differences in CD4 response or short term virologic response [25]. Racial differences were found in virologic response after 9 months however, poorer responses in the African cohort were thought to be attributable to lower adherence in this group.

In accordance with these prior studies, our study also showed no racial differences in virologic or immunologic responses to HAART. In other words, both Aboriginal persons and non-Aboriginal persons in our group on average achieved a typical response to HAART. This is characterized by a rapid decrease in HIV viremia to undetectable levels and a gradual increase in CD4 cell count to levels approximating those in uninfected individuals [42].

Survival has also been used to examine potential racial/ethnic differences after initiation of HIV treatment. Prior studies have found an increased mortality risk for HIV-infected racial/ethnic minority groups who had non-significant differences in clinical management or HIV-treatment [43,44]. In an examination of trends in survival amongst men who have sex with men and who were diagnosed with AIDS during the HAART era, declines in deaths were smaller among racial minorities (black, Hispanic, Asian/Pacific Islanders, American Indian/Alaskan Native) compared with whites [45]. These studies indicate that race/ethnicity has an unfavorable effect on mortality risk, however, none of these studies directly measured the effects of HAART.

This study has a number of limitations. Our measure of a history of IDU was self- or physician-reported. Because injection drug use is a stigmatized behaviour, a history of IDU may be underreported. A history of IDU was also a baseline measure that would not account for injection drug users who might have become abstinent during follow-up [46]. Secondly, although using refill compliance as a measure of adherence has been previously validated, [37,46-49] it may not account for individuals who received their medication but did not actually take it. In the two situations described previously, the misclassification present in the variables history of IDU and adherence could potentially bias the associations between Aboriginal status and the three outcomes of interest. The analyses for mortality and CD4 cell recovery showed a similar association between Aboriginal status in both unadjusted and adjusted regressions. The difference between the coefficients for aboriginal in the univariate and multivariate analyses ranged from 0.10 (cell recovery analysis) to 0.25 (mortality analysis), which indicates that misclassification bias or residual confounding did not influence these results. Note that for the analysis of viral suppression the coefficient for Aboriginal status, though not statistically significant, changed the direction of association. We conducted a more detailed analysis for this outcome to assess the effect of confounding and misclassification bias on the relationship of Aboriginal status and viral suppression. We observed that adherence was the strongest confounder in this analysis, since the hazards for aboriginal status across the levels of adherence were substantially different, ranging from 0.58 (95%CI: 0.37–0.92) for <95% adherence to 1.34 (95%CI: 1.01–1.78) for ≥95% adherence. Therefore, the coefficients for aboriginal just controlling for adherence and the coefficient shown in Table 3 changed by 0.05, which shows that our results were not influenced by misclassification bias or residual confounding.

The all-cause mortality rate in this cohort was associated mainly with HIV disease resulting in infectious and parasitic diseases (48%) and injuries due to intentional self-harm (19%). In addition to Aboriginal status, there were several potential clinical and socio-demographic confounders taken into account in our study. However, we did not control for the effect of co-morbidity (e.g., psychiatric illnesses), co-infections with hepatitis C, anemia, and other lifestyle (e.g., cigarette smoking) characteristics known to be related to HIV disease progression [13,14,16-18,21,48]. These factors could have influenced our results by biasing our results through residual confounding. We believe that controlling for other socio-demographic and lifestyle variables other than age, sex, income, education and history of IDU would not change our results. In our cohort we do not have data on co-infections collected longitudinally, other than hepatitis C. If we decided to include information on hepatitis C in our study, all our analyses and objectives would have to be changed dramatically mainly because of two reasons: (1) being infected with hepatitis C would not be considered a confounder variable, but it would be a factor influencing the definition of our study population; (2) we would need more study subjects to study the association of being aboriginal and clinical outcomes separately according to three distinct disease groups: (i) HIV positive, HCV negative; (ii) HIV negative, HCV positive; and (iii) HIV positive, HCV positive.

Finally, the sample size in our study was small. There were 569 participants with unknown Aboriginal status that were not included in our analyses. Like in all observational studies collecting information on socio-demographic characteristics, there is always a chance for missing information. To date several studies dealt with missing information via assumptions about the missing data or via imputation techniques for missing data. In this study, we decided to not include participants with missing information on the exposure of main interest. As reported in our results, there were significant demographic and clinical differences between participants and non-participants. Therefore, we recommend caution when interpreting the results from our analyses and extrapolating to other minority populations.

Our study highlights the need for continued research on medical intervention for HIV-infected Aboriginal persons, in particular to determine if providing services for Aboriginal drug users to address their addictions can improve survival after the initiation of HAART. Understanding the mechanism by which such health care disparities exist by determining what other aspects of being Aboriginal increase their risk of mortality after initiating HAART can provide potential targets for intervention in this vulnerable population. Results could be further extrapolated to the understanding of health care inequalities amongst other marginalized populations.

## Competing interests

The author(s) declare that they have no competing interests.

## Authors' contributions

**VDL: **design, statistical analysis, write-up. **PK: **design and write-up. **AP: **design and write-up. **SB: **design and statistical analysis. **TK: **design and write-up. **DM: **design and write-up. **MD: **design and write-up. **JSGM: **design, and write-up. **RSH: **design, data gathering, and write-up.
